# Cultured beef: from small biopsy to substantial quantity

**DOI:** 10.1002/jsfa.10663

**Published:** 2020-07-27

**Authors:** Lea Melzener, Karin E Verzijden, A Jasmin Buijs, Mark J Post, Joshua E Flack

**Affiliations:** ^1^ Mosa Meat B.V. Maastricht Netherlands; ^2^ Department of Physiology Maastricht University Maastricht Netherlands; ^3^ Axon Lawyers Amsterdam Netherlands

**Keywords:** cultured meat, cultured beef, biopsy, regulatory, donor cattle, stem cell isolation

## Abstract

Cultured meat is an emerging technology with the potential to solve huge challenges related to the environmental, ethical, and health implications of conventional meat production. Establishing the basic science of cultured meat has been the primary focus of the last decade but it is now feasible that cultured meat products will enter the market within the next 3 to 4 years. This proximity to market introduction demands an evaluation of aspects of the cultured meat production process that have not yet been outlined or discussed in significant detail. For example, one technological approach for the production of cultured meat uses adult muscle stem cells, the limited proliferative capacity of which necessitates repeated collection of tissue samples via biopsies of living donor animals. The selection of donor animals and the details of biopsy processes must be optimized, as this is a key bottleneck in the cultured meat production process. The number of stem cells harvested from a biopsy, together with their proliferative capacity, determines a ‘multiplicity factor’ achieved by a cultured meat production process, thus dictating the reduction in number of animals required to produce a given quantity of meat. This article considers potential scenarios for these critical upstream steps, focusing on the production of cultured beef as an example. Considerations related to donor selection and details of the biopsy process are discussed in detail. The practicalities of various scenarios for cultured beef production, the health of donor animals, and regulatory issues associated with the safety of cultured meat for consumers are also considered. © 2020 The Authors. *Journal of The Science of Food and Agriculture* published by John Wiley & Sons Ltd on behalf of Society of Chemical Industry.

## GLOBAL CHALLENGES IN THE MEAT INDUSTRY

Global meat consumption has risen dramatically in recent decades, and is predicted to increase by a further 73% by 2050.^1^, ^2^ The production of 1 kg of beef requires over 15 000 L of water, around 40 m^2^ of land, and produces 300 kg CO_2_ equivalents.^1^ Globally, one third of all available land globally is used for agricultural purposes, of which the vast majority is used as pasture for livestock. Furthermore, 30% of all crops are fed to animals, which is hugely inefficient due to low feed conversion ratios (FCRs). In cattle, the most inefficient species, the FCR is approximately 6:1.^2^, ^3^, ^4^ This increase in the demand for meat will also result in an intensification of existing conflicts over food and water. Major public health concerns, including the ongoing COVID‐19 virus pandemic, have been linked to patterns of human meat consumption.^5^, ^6^, ^7^ Hence, more efficient and less environmentally damaging systems of production are urgently required to meet global meat demand by 2050 and beyond.

**Figure 1:**
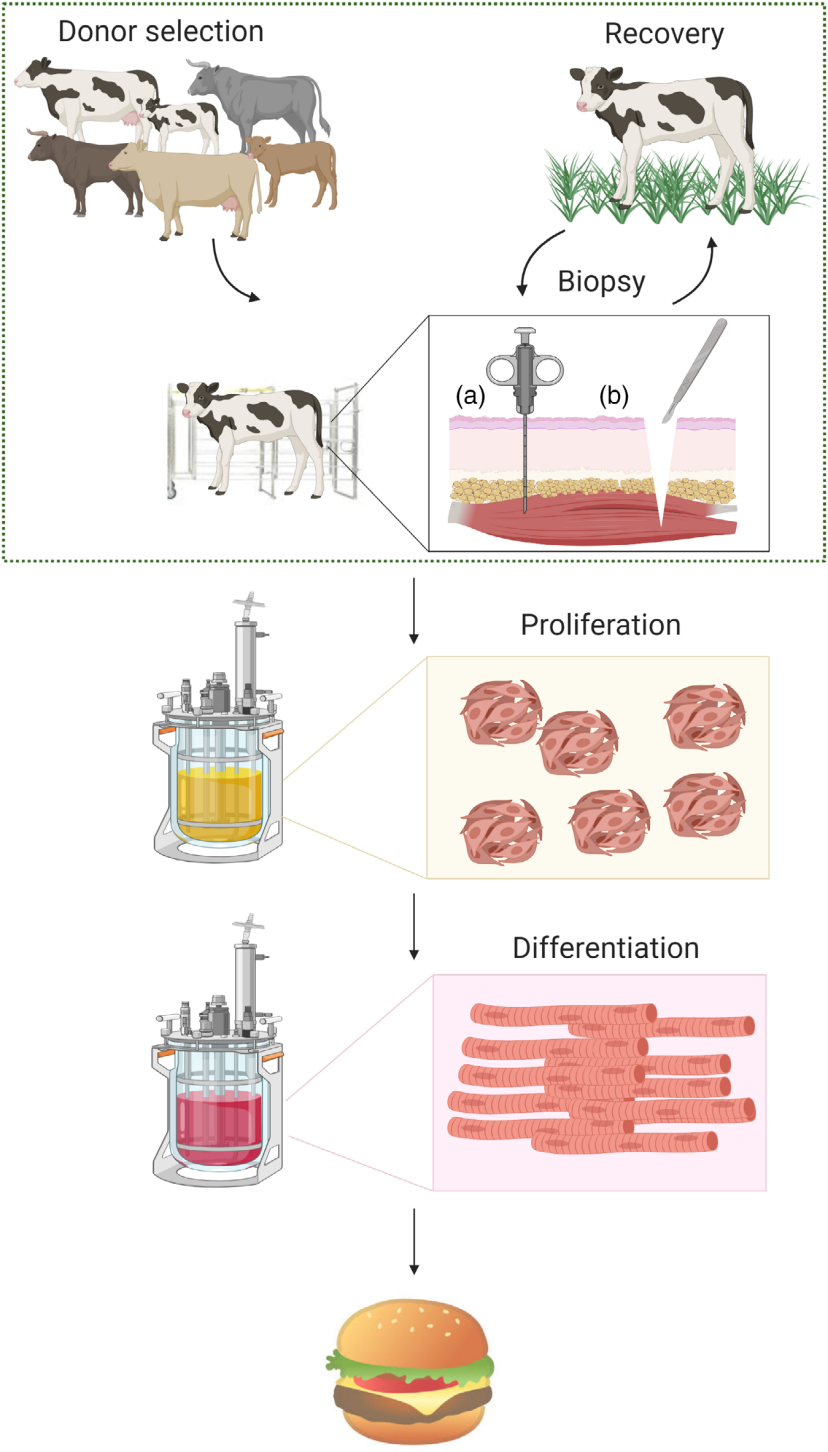
Overview of cultured beef production process. The boxed region indicates the topic areas discussed in this article. The inset panel compares methods for (a) needle biopsy, and (b) incision biopsy for the harvesting of muscle tissue.

This pressure on traditional (also henceforth referred to as ‘conventional’) meat production from livestock has triggered the development of cultured meat (also referred to as ‘cell‐based’ or ‘*in‐vitro’* meat) technologies, which have the potential to be vastly more resource efficient, sustainable, and animal friendly.^8^, ^9^, ^10^ Although several technological approaches are being explored, the simplest involves the isolation of adult stem cells from a donor animal, and the use of cell culture methods to expand these cells to large numbers in bioreactors.^8^, ^9^ Tissue engineering methods are then used to differentiate the expanded stem cells into muscle and fat tissue, which are used to generate cultured meat products that closely mimic traditional meat (see Fig. 1). Based on lifecycle analysis, it has been shown that the introduction of cultured meat technologies could reduce the environmental impact of meat consumption by up to 90%.^11^, ^12^ Crucially, such technologies could reduce the number of cattle worldwide by many orders of magnitude.

As a new technology being introduced into the food production system, the manufacturing process of cultured meat will need to be described and analyzed in detail, and guided by novel regulation. In this article, we focus on the initial steps of this process; the harvesting of stem cells from donor animals via tissue biopsies (see Fig. 1, boxed area). These crucial upstream steps require careful consideration to maximize process efficiency, afford animal comfort, and ensure consumer safety. We have focused on biopsies in cattle for the production of cultured beef as an illustrative example, but while species‐specific considerations will certainly apply, the general principles are likely to be transferable. Specific considerations relating to donor animal selection, biopsy technique, animal care and handling and quality management are discussed, and key areas for scientific and political decision making are highlighted.

## OPTIMIZED STEM‐CELL DONORS FOR CULTURED BEEF

The first variable in any cultured meat production process is the stem‐cell donor animal. Donor selection can dramatically affect the efficiency of the entire process, and identifying the defining characteristics of a good cell donor is thus critical. There are two key parameters that must be optimized for: the yield of stem cells per mass of tissue, and the longevity of those stem cells (i.e. the number of populations they can undergo, whilst retaining the ability to differentiate to form mature tissue for meat production). Together, these parameters determine a multiplicity factor; specifically, the mass of cultured meat that can be produced from a given mass of starting tissue. As the main component of meat products is muscle tissue (generally around 87.5% muscle and 12.5% fat for a standard hamburger),^13^ and there are many potential starting cells types for fat tissue production, this discussion of donor characteristics will focus on the harvesting efficiency and quality of muscle‐specific stem cells (also known as, and henceforth referred to as, ‘satellite cells’).^14^ However, it is important to note that similar considerations will apply for the selection of donor animals for cultured fat production.

Numerous characteristics of the donor cattle can affect the yield and quality of satellite cells. The age of the donor is an obvious and crucial parameter that must be optimized; several groups have reported that satellite cell content of muscle decreases significantly with increasing age.^15^, ^16^, ^17^, ^18^ A rapid decrease in number of satellite cells occurs during the first months after birth; in humans it was found that satellite cell content decreases by 30‐fold between the ages of 0 and 18 months.^18^ In neonates satellite cells can represent up to 30% of all nuclei,^16^ but this figure decreases to 2% in adult organisms.^15^ Whilst these figures may not be directly comparable between species, this clear trend is reproduced across all tested species. Moreover, as satellite cells from younger animals have experienced fewer mitotic cell divisions, one might also expect them to retain their differentiation capacity for a longer proliferative period.^19^, ^20^, ^21^ Sex may also influence the yield and quality of satellite cells; these stem cells express androgen receptors, stimulation of which may trigger cell proliferation, whilst androgens themselves have been shown to be positively correlated with muscle strength and size in both agricultural animals and humans.^22^ Furthermore, there are likely to be differences in the yield and quality of satellite cells from different cattle breeds. Coles *et al*. (2015) identified differences in proliferation rates between satellite cells from Angus, Hereford, and Wagyu cattle, although they did not detect significant differences in the myogenic nature of these cells, as measured by expression of MyoD.^23^ Further studies are ongoing in this area.

The site of biopsy with respect to anatomically distinct muscles or muscle groups may also be highly relevant. Different muscles have varying muscle fiber type composition, which can be correlated with the ratio of satellite cell nuclei to myonuclei; Type I (‘slow‐twitch’) fibers have been shown to contain higher numbers of satellite cells when compared to Type II ('fast twitch').^13^, ^14^, ^15^, ^16^ In cattle, muscles belonging to the chuck contain primarily Type I fibers, while those of the round contain predominantly Type II.^24^ Fiber composition can also be influenced by husbandry conditions; Vestergaard *et al*. (2000) showed that the fiber constellation between animals from intensive and extensive husbandry conditions varies as a result of the composition of the feed (more precisely, as a result of the amount of roughage).^25^ In animals raised in extensive husbandry conditions the proportion of Type IIB fibers in all examined muscles was reduced when compared with intensively reared animals, whilst the proportion of Type I fibers was increased.^25^

Accordingly, an optimal satellite cell donor may theoretically be a young bull, kept in extensive holding conditions with a roughage‐based diet, in which the biopsy is taken from the chuck at the age of a few months. However, further studies will need to be undertaken to confirm the influences of these parameters on the yield and quality of satellite cells. Interesting, alongside satellite cells, other stem cell populations in muscle tissue have also been described. Fibroadipogenic precursors (FAPs) are multipotent precursors with the potential to differentiate into fibroblasts, adipocytes, and potentially also into osteoblasts and chondrocytes.^26^, ^27^, ^28^, ^29^ Using FAPs as starting cells for fat production could allow the generation of both muscle and fat tissue from a single muscle biopsy. How the donor characteristics discussed in this section correlate with the yield and quality of FAPs from a muscle biopsy is, as of yet, unknown.

## BIOPSY TECHNIQUES AND PROTOCOLS

Although satellite cells (and other stem cells) can be obtained from the tissue of freshly slaughtered animals, the starting material for cultured beef production will ideally be obtained through muscle (and fat) tissue biopsies (for some other types of cultured meat, such as avian or fish species, the possibility to sample cells from fertilized eggs may obviate the need for biopsies). The site of biopsy has been discussed previously, but the details and protocols for the process of biopsy‐taking also require careful consideration. Tissue biopsying is a standard procedure in human and veterinary medicine, most commonly performed via a needle biopsy (see Fig. 1, biopsy insert (a)). This procedure is relatively quick, and has the advantage of causing minimal stress and trauma to the subject. Disadvantages, however, include the limited size of the biopsy (approximately 0.5 g), and the blind nature of sample taking, potentially leading to failures where samples of the desired tissue type or quality are not obtained. A second technique for the taking of biopsies, which may be preferable for cultured meat applications, is *via* a small incision (Fig. 1, biopsy insert (b)). This method is more invasive than a needle biopsy, but the sampling is more controlled and greater amounts of material can be obtained (sample size with this technique, although flexible, can be up to 15 g).^30^ For both techniques, an optimal protocol will likely involve immobilization of the donor animal in a treatment cage, sedation with xylazine (or equivalent), and local anesthesia with lidocaine/adrenaline to minimize pain and stress to the animal and ensure safety of the veterinarian, thus improving the quality of tissue samples.^30^

To minimize the number of animals required to produce a given mass of cultured beef, it will be desirable to maximize the number of biopsies taken from each donor animal. Parameters such as the degree of discomfort and stress felt by the animal (and thus the effect on animal welfare), and the length of time required for tissue regeneration, must be taken into account. Ethical evaluations will have to be made to define the maximum number of biopsies taken per session, and the maximum number of sessions per animal. When assessing these considerations it will be helpful to determine which parts of the procedure are most stressful for the animal. The current notion is that the actual act of sedation and immobilization in a cage will represent the biggest source of discomfort, thus suggesting that a larger number of biopsies per session may be preferable to a larger number of sessions.

Studies testing the effect of tissue biopsies on animal welfare have been carried out in a number of different settings. Mølgaard *et al*. (2012) reported that taking liver biopsies in dairy cattle had an observable effect on the behavior of the animals for 19 h after the procedure, consistent with pain being experienced.^31^ No pain‐associated behavior patterns could be observed 20 h after sampling. No post‐procedure analgesics were used in this study, and the authors suggested that inclusion of analgesics and an additional improvement in the anesthetic protocol could further reduce the pain experienced.^31^ Muscle biopsies represent a significantly less invasive procedure. A study on mammary gland biopsies in cattle showed that it is possible to take repeated biopsies, from slightly different positions, with an interval of 3 weeks.^32^ Samples that were taken in this study had a mass of around 800 mg, with tissue showing full healing within 7 days.^32^ Assuming that the biopsy will be taken with a needle, the discomfort for the animal will likely not be greater than the taking of a blood sample, whilst in case of an incision biopsy, the level of discomfort will likely be greater, although still not substantial.

Based on the studies mentioned above, and current practices in farming, one cautious first approach for the protocol and frequency of muscle biopsies for cultured beef could be as follows: multiple (up to four) biopsies are taken from each donor animal in a single session every 3 months, using a needle biopsy technique, each providing around 500 mg of tissue. The animal is treated with an analgesic to minimize post‐procedural pain and discomfort. Subsequent biopsies can be taken from slightly different locations within the same muscle (to make sure the fiber quality is still maximized, whilst avoiding the collection of fibrotic scar tissue formed as a result of prior biopsies). Further studies will be required to optimize these protocols and procedures in light of veterinary and animal experience.

## CULTURED BEEF DONOR FACILITIES

It is important to consider the required scale of such an operation when contemplating what cultured meat donor animal facilities might consist of when this technology becomes viable on a commercial scale. As previously introduced, a critical parameter in such calculations is the multiplicity factor (the amount of cultured meat that can be produced from a given starting mass of biopsied tissue). This depends on stem‐cell yields, but also on the number of cell doublings that these cells can achieve whilst maintaining their capacity to differentiate into muscle (or fat) tissue. This is empirically limited to 50 doublings for non‐immortalized primary cells (although for some cell types, reported numbers of doublings have been higher).^33^

Global beef consumption in 2013 was approximately 70 million tons.^34^ A satellite cell ‘quality’ of 35 population doublings could correspond to a multiplicity factor of roughly 10^7^ (equating to production of 5000 kg of cultured beef from a single 500 mg biopsy). One biopsy could thus replace the slaughter of 20 cattle. If 20 such biopsies could be taken per animal, a single donor could replace 400 cattle over its lifespan. If 50 population doublings could be achieved, due to the exponential nature of the proliferation process the multiplicity factor would increase to over 10^11^ so that a single biopsy could generate enough meat to replace 13 million cattle. This could theoretically reduce the required number of cattle held globally from over 1 billion to less than 100.^35^ Improvement of cell proliferation conditions is thus clearly a crucial hurdle that requires further optimization in order to minimize the required number of donor animals.

However, if such high cattle replacement rates could indeed be achieved, the number of animals required will no longer be determined by tonnage of meat production, but by other factors such as the minimum viable population size of a herd. The minimum viable population was described by Franklyn (1980) as 500 animals (250 male and 250 female animals, all involved in reproduction).^36^ Once the sex ratio shifts, the minimum number of individuals of one sex is 126, whilst the number of individuals of the other increases significantly to over 20 000. This is typical practice, where the cows in a herd significantly outnumber the bulls. If one compares these population sizes with the number of agriculturally used cattle in 2019, which is approximately 1 billion,^35^ a population size of 20 000 animals would still represent a massive reduction of the number of cattle in livestock production, and reduce global meat‐cattle‐associated greenhouse gas emissions to negligible levels. The geographical spread of herds, and the maintenance of different agriculturally relevant cattle breeds will be other considerations when designing a future donor system for cultured beef.

Besides meat production, the global markets for milk and dairy products (as well as leather) will necessitate the keeping of cattle. Worldwide, over 250 million cows are kept for the production of approximately 600 million tons of milk every year. Numerous research groups and companies are already focusing on the development of cultured milk and other diary products.^37^ Nevertheless, the global number of cattle will only be significantly reduced if both cultured beef and milk can be produced successfully.

A further consideration is the fate of donor cattle once they are no longer able to serve as effective stem cell donors. An ethical position may be that the animals can continue their lives until they die from natural causes. However, from an environmental and efficiency perspective, it would be more logical to slaughter the animals and either harvest all of their remaining satellite cells (producing huge quantities of cultured meat), or produce traditional meat from the carcass. Future scenarios will depend on the likely continued existence of a conventional meat market, alongside an expanding market for cultured meat. If, for instance, the conventional market will continue to have a share as small as 10%, the number of cows needed to produce that volume will still far exceed the number needed to produce the other 90% through cultured meat. In such a scenario, it may be reasonable to slaughter the animals at an age where they are no longer considered productive as donor animals. These considerations may also have implications for cultured meat inspection processes.

## CULTURED BEEF PRODUCTION IN 2030: A CASE STUDY

Given the nascency of cultured meat as a field, and uncertainties over the pace of technological development and adoption, there are a wide variety of potential scenarios for the integration of cultured meat into existing livestock husbandry systems and the wider meat market over the next decade. These scenarios could range from a small number of ‘super farms’ containing many thousands of donor cattle, to many smaller farms where donor herds are thinly spread over regions to supply small communities or cities. In any of these scenarios, a combination of cultured meat production with ongoing conventional meat production can be considered.

In this perspective we present a potential vision for the integration of cultured beef into the meat market of small regions in the latter scenario, using the Dutch city of Maastricht as a case study. Maastricht, a city in the south of the Netherlands, has around 120 000 inhabitants. In the Netherlands, average annual per capita meat consumption is approximately 75 kg, of which 16.7 kg is beef and veal.^38^, ^39^ Approximately 2 million kg of beef is thus consumed in Maastricht *per annum*. To produce this amount of meat via a conventional production system, assuming an average carcass muscle weight of 250 kg, at least 8000 cattle need to be slaughtered each year. However, if 5000 kg of cultured beef could be produced from a 500 mg biopsy (and taking into account various assumptions discussed in previous sections), only around 50 cattle would be needed to meet the beef demand of Maastricht for up for 2 years.

As discussed, a minimum of 500 animals are necessary to ensure a viable population without inbreeding issues, but as artificial insemination is a common technique used in the livestock industry, several small herds that function as donors for particular regions can be treated as one population with an effective herd size of 500. In a scenario with a multiplicity factor of 10^7^, we foresee that this might result in the collaboration of Maastricht with around 10 other towns or small cities with a single donor facility for cultured beef production. If we consider a multiplicity factor of 10^11^, a single donor facility could supply cultured beef to all of Europe. This multiplicity factor, combined with the other features of cultured beef production discussed here, thus has a huge impact in determining how the future of this industry will be organized physically.

## SAFETY AND LEGALITIES OF CULTURED MEAT PRODUCTION

### Regulatory frameworks for meat inspections

Alongside the scientific and practical advances required, for cultured meat to enter the market specific new regulations and inspection practices may need to be added to the existing frameworks in place for the conventional meat industry. In the EU, cultured meat (either marketed as such or as an ingredient) is defined under the General Food Law (GFL) (Regulation (EC) No 178/2002) as a food product just like conventional meat, and authorization is therefore required to ensure that it is safe for consumption.^40^ From a legal standpoint, it is expected that cultured meat will make its way to the market in the EU under the Novel Food Regulations (NFR) (Regulation (EC) No 2015/2283) because it was not consumed to a significant degree by humans in the EU prior to 15 May 1997.^41^ The general principle of the GFL, which applies at all stages of production, is that food products shall not be unsafe.^40^ In the field of meat production, food safety is first achieved by applying the hygiene standards laid down in the Hygiene Regulations ((EC) No 852/2004^42^ and 853/2004^42^). Second, official controls are warrantied on the basis of the New Official Controls Regulation 2017/625, including controls on the application of good hygiene practices and the Hazard Analysis and Critical Control Points (HACCP) principles.

After a number of food and feed crises in the 1980s and 1990s, food law has been largely harmonized in the EU with the introduction of the GFL.^40^ Reference is made to the transmissible spongiform encephalopathy (TSE) crisis, which emerged due to the feeding of cattle with animal proteins (i.e. bone meal produced from leftovers of the slaughtering process), and to the dioxin crisis, which occurred due to chickens being fed feed contaminated with industrial oils. The European Commission (EC) is the driver to overlook food safety, supported by various advisory bodies such as the European Food Safety Authority (EFSA). The EFSA has a statutory role in certain authorization procedures, like the novel food procedure, and can also be requested by the Commission to render scientific opinions regarding specific topics. EU food law is mostly embodied in regulations that are directly applicable in the member states, although additional national standards can apply. Actual enforcement of food safety, as well as official controls based on EU requirements, is the responsibility of the competent authorities of each member state.

### Inspections in conventional meat production

**Figure 2:**
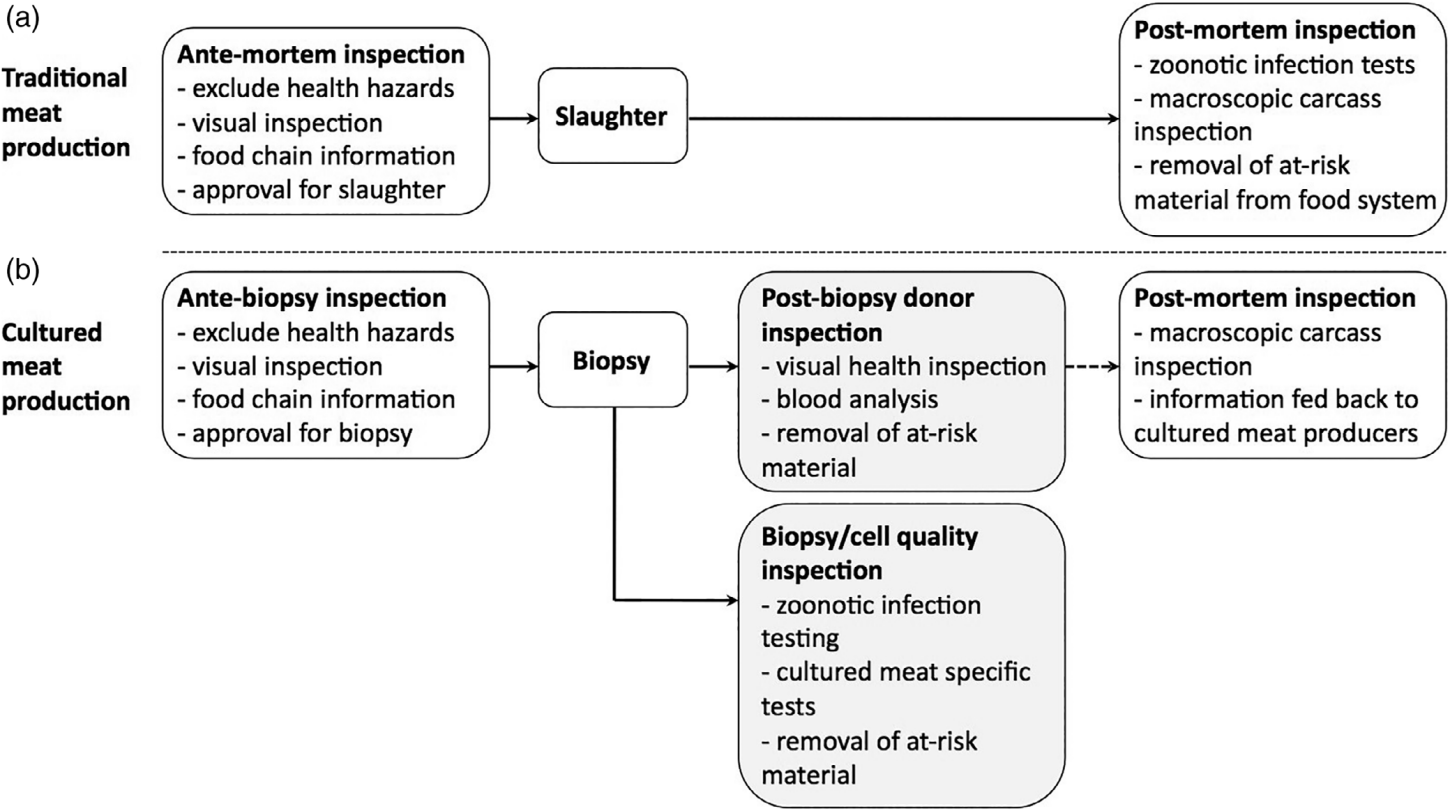
Comparison of inspection processes for traditional and cultured meat production.

In conventional meat production, food safety is assured by the execution of ante‐mortem inspection of living animals, and post‐mortem inspection of carcasses and organs (Fig. 2(a)). The scope of these inspections includes the monitoring of zoonotic infections and the detection of certain animal diseases with the purpose of assessing whether the meat is fit for human consumption in general, and to address a number of specific hazards, such as TSE, contaminants, and residues of veterinary drugs.^43^ Ante‐mortem inspections are performed by an official veterinarian (see Article 18.2 (a) New Official Controls Regulation,^44^ which replaces the Old Official Controls Regulation^45^), and veterinarian approval is required prior to the slaughter of the animal, based on mandatorily submitted food chain information (see Annex II, section III, point 7 to Regulation (EC) 853/2004).^42^ Post‐mortem inspections are likewise carried out by an official veterinarian.

Ante‐mortem inspection is defined as the verification of animal health, including, where appropriate, the clinical evaluation of the animal prior to slaughtering and the verification of food‐chain information applicable to the animal (see Article 17 (c) New Official Controls Regulation).^44^ According to EFSA,^43^ ante‐mortem inspection is usually conducted by visual observation of animals in motion: on arrival, i.e. during unloading, and, in the case of extended lairaging, just prior to sending them from lairage to slaughter. The rationale for such inspections lies in the fact that the animal should be clean and healthy for acceptance onto slaughterhouse premises (Regulation (EC) 853/2004).^42^

Post‐mortem inspection, meanwhile, consists of verification of compliance with requirements applicable to carcasses, safe removal of specified risk material, and the health and welfare of animals. As with ante‐mortem inspection, the Hygiene Regulations^46^ and the new Official Controls Regulation^44^ do not detail how the ‘health and welfare of animals’ should be assessed once the animal has been slaughtered. The Old Official Controls Regulation has specified, in Annex I, Section IV, a number of post‐mortem inspection requirements for bovine animals under and above 6 weeks old, amongst others.^45^ These requirements are no longer listed in the New Official Controls Regulation.^44^ The EFSA states that post‐mortem examination with respect to bovine animals is conducted macroscopically at multiple inspection points in the slaughter line. This may be performed by simple visual inspection, or by palpation or incision.^43^

### Proposed inspections for cultured meat production

For cultured meat production, we envisage that inspections prior to biopsy taking will be similar to ante‐mortem inspection performed for conventional meat (see Fig. 2(b)). This means, for example, that biopsies should only be taken from animals that are declared healthy upon visual inspection by a veterinarian. That declaration would simultaneously confirm that no health hazards are present in the food‐chain information relating to the respective donor animal. As in conventional meat production, this food‐chain information should cover vital information, including the animal's health status, any veterinary medicinal products administered to the animal at stake, under specification of the dates of administration and the occurrence of diseases that may affect the safety of the meat (see Annex II, section III, point 3 of Regulation (EC) 853/2004).^42^

In cultured meat production, the animal from which a biopsy is taken will not die as a result, and immediate post‐mortem inspection is thus not directly applicable. The main purpose of post‐mortem inspections is detection of specific meat‐borne pathogens, in particular those of a zoonotic nature such as tuberculosis and brucellosis, and to detect any fecal contamination.^43^ In a cultured meat setting, the former can be achieved by blood analysis of the donor animal, and the latter by lab analysis of the sample itself. Although the inspection process will therefore not be identical, we do not envisage that it will be challenging to achieve equal or superior levels of food safety in a cultured meat process. In this context, it is also worthwhile to note that the EFSA seems to consider the effect of post‐mortem inspections to be limited. This is caused by the fact that the majority of patho‐anatomical abnormalities found at post‐mortem examination are not zoonotic hazards *per se*. The results obtained from macroscopic post‐mortem testing were sometimes already obtained by ante‐mortem inspections. Moreover, it may be that donor animals are subsequently slaughtered for conventional meat production, in which case cultured meat business operators may want to know if post‐mortem inspections reveal any safety and / or quality concerns, especially when the animal is slaughtered soon after the sample has been taken.

In some areas, specific safety inspections for cultured meat processes may be required that differ from those employed in the conventional meat industry. For example, levels of particular medium components in final meat products may have to be assessed if they are not usually present in conventional meat. Alternatively, the proliferation of stem cells in culture for many population doublings could lead to the accumulation of genomic alterations that might have negative implications for food safety. Although this is an unlikely scenario, it demonstrates the importance of the NFR in guiding the market introduction of cultured meat products. Specific tests, such as DNA genotyping, could be employed to ensure compliance of cultured meat products within strictly defined safety limits.

In conclusion, in our opinion the above has shown that biopsy‐taking for cultured meat production from donor animals that are not slaughtered can fit into the current regulatory system for meat inspections when applied with minimal adjustment. Moreover, the opportunity to analyze the biopsied material (and the harvested stem cells) with sophisticated and sensitive laboratory techniques will likely provide ascertainment of a disease‐free status at the start of the cultured meat production line that is at least as accurate as in conventional meat production. Nevertheless, discourse between cultured meat producers and regulatory authorities will be required prior to commercial adoption of cultured meat on any scale.

## OUTLOOK

Cultured meat is a highly promising technology, with the potential to address existential problems for humanity, in terms of the environmental, ethical, and health implications of our current meat industry. Here we have focused on cultured beef, where studies are still ongoing to determine optimal methods for obtaining the starting material for production, and to select the best donor cattle based on factors such as age, gender, breed, and known muscle composition. At this point, scientific and practical barriers to the commercialization of cultured beef remain. Nevertheless, if the current state of technology in the cultured meat field can be successfully scaled up, a multiplicity factor of 10^7^ would allow a reduction in the number of cattle required for global beef production by a factor of 400. With further improvements in the technology (crucially, achieving greater numbers of stem cell population doublings) it might theoretically be possible to meet the annual global demand for meat from a single biopsy. At such a point, the number of cattle required for beef production would be determined by factors such as healthy herd sizes and geographical spread. What the future of the meat industry will look like over the coming decades, with respect to the roles of cultured and traditional meat production, remains hugely unknown. In all future scenarios, livestock systems will need to be significantly adapted if we are to meet global meat demands while meeting ratified climate targets. In any scenario where cultured meat plays a major role, the subsequent reduction in animal numbers could ensure that globally available land be made available directly for food production. As cultured meat is considered a novel food, novel regulations and regulatory oversight systems may have to be developed in parallel with the technology to ensure its consistent quality and safety. That said, the regulatory framework for conventional meat in the EU can be applied to cultured meat by analogy, and simply needs to be put to the test by the first applicant for a novel food authorization.
